# A Review of L-Asparaginase Hypersensitivity in Paediatric Acute Lymphoblastic Leukaemia Patients with Regard to the Measurement of Anti-Asparaginase Antibodies and Their Genetic Predisposition

**DOI:** 10.21315/mjms2023.30.5.4

**Published:** 2023-10-30

**Authors:** Yan Qi Tan, C-Khai Loh, Suzana Makpol

**Affiliations:** 1 Department of Paediatrics, Faculty of Medicine, Universiti Kebangsaan Malaysia Medical Centre, Kuala Lumpur, Malaysia; 2 Department of Biochemistry, Faculty of Medicine, Universiti Kebangsaan Malaysia Medical Centre, Kuala Lumpur, Malaysia

**Keywords:** L-asparaginase, hypersensitivity, immunogenic, acute lymphoblastic leukaemia, antibodies, genetic polymorphisms

## Abstract

L-asparaginase is effective as part of the first line childhood acute lymphoblastic leukaemia (ALL) treatment regimen but suffers the risk of antibody production causing immune-mediated sequelae. This article aimed to describe the clinical implication of L-asparaginase hypersensitivity and review the types of antibodies and genetic polymorphisms contributing to it. Clinical or subclinical L-asparaginase hypersensitivity may lead to suboptimum therapeutic effect and jeopardise the clinical outcome in ALL children. Anti-asparaginase antibodies immunoglobulin (Ig)G, IgM and IgE were identified in the L-asparaginase hypersensitivities. Enzyme-linked immunosorbent assay (ELISA) is commonly used to quantify the IgG and IgM levels. The role of IgE in mediating L-asparaginase hypersensitivity is contradictory. Moreover, the presence of antibodies may not necessarily correlate inversely with the L-asparaginase efficacies in some studies. Patients with specific genetic variants have been shown to be more susceptible to clinical hypersensitivity of L-asparaginase. With the advance of technology, gene polymorphisms have been identified among Caucasians using whole-genome or exon sequencing, but the evidence is scanty among Asians. There is lack of pre-clinical study models that could help in understanding the pathophysiological pathway co-relating the gene expression and anti-asparaginase antibody formation. In conclusion, future research studies are required to fill the current gap in understanding the immune mediated reactions towards L-asparaginase upon its administration and its potential impact to the disease outcome.

## Introduction

Acute lymphoblastic leukaemia (ALL) is defined as a malignancy that originates from the failure of lymphoid cells maturation and uncontrolled proliferation of lymphoid progenitors in one’s body. In turn, these immature lymphoblasts outnumber the normal haematopoietic cells and occupy the bone marrow or metastasise to extramedullary sites such as the liver, spleen and lymph nodes through blood circulation (1). The clinical manifestations are the result of these immature lymphoblasts replacing the normal haematopoietic cells in the bone marrow, presented as anaemia, leukocytopenia, and thrombocytopenia and even bone marrow failure, in patients (2, 3).

ALL is the most prevalent type of cancer among children. Studies showed that ALL constituted approximately 25% of the total malignancy cases in this population (below 15 years old) (4). It was estimated that more than 54,000 new childhood ALL cases emerge per annum in Asia (5). The 5-year survival rate of childhood ALL in developed countries has surpassed 90% (6). There is a significant disparity between estimated 5-year survival rates for childhood ALL in Asian countries, ranging between 44.5% and 80% (4), possibly due to the varying extents of availability of medical resources and the lack of a proper diagnostic method in some low-income Asian countries.

**Figure 1:**
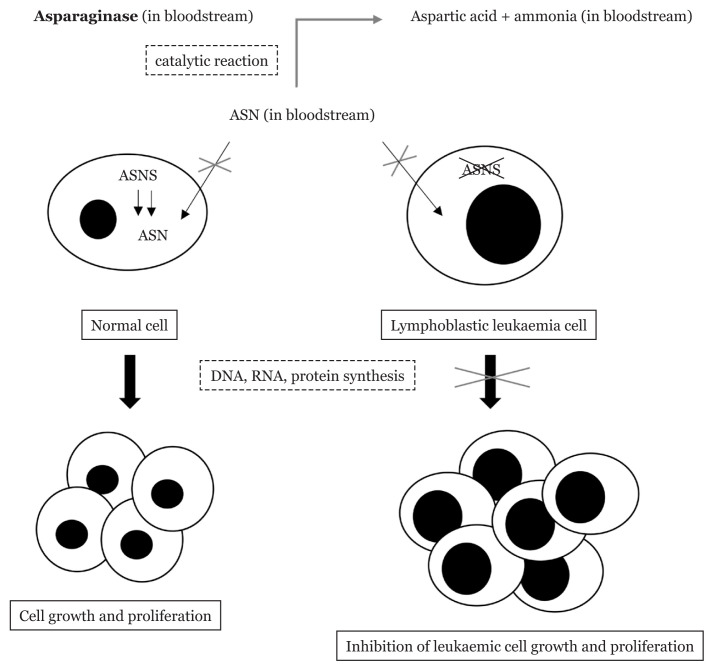
Mechanism of action of L-asparaginase in selective therapy of ALL Notes: ASN = asparagine; ASNS = asparagine synthetase; DNA = deoxyribonucleic acid; RNA = ribonucleic acid

L-asparaginase, an enzymatic chemotherapeutic drug, has been introduced as the first line treatment regimen for childhood ALL. In the early 1970s, it was evidenced in clinical trials that paediatric patients who received treatment protocols that included L-asparaginase had better clinical outcomes compared to those who did not receive the L-asparaginase treatment (7, 8). The mechanism of action of L-asparaginase in removing the blast cells is as shown in Figure 1. L-asparagine is a non-essential amino acid in the human body. It is synthesised by the normal cells for use in the synthesis of DNA, RNA and proteins necessary for growth (9). Unlike normal cells, the blast cells do not have the capability of producing L-asparagine in sufficient amounts owing to the absence of adequate asparagine synthetase enzyme (10). Hence extracellular sources of L-asparagine are essential for these tumour cells to sustain their excessive proliferation (9). By catalysing the hydrolysis of L-asparagine in the bloodstream into L-aspartic acid and ammonia, L-asparaginase reduces the plasma level of L-asparagine in ALL patients. This results in the deficiency of L-asparagine required for the uncontrolled growth processes, eventually causing apoptosis of these lymphoblastic leukaemia cells (9).

In general, the commercially and clinically available L-asparaginase preparations for childhood ALL are derived either from the bacteria *Escherichia coli* or *Erwinia chrysanthemi*. The *E. coli* L-asparaginase is produced either in native or the pegylated form, the latter has a covalently attached monomethoxypolyethylene glycol polymer. The pegylated *E. coli* L-asparaginase has been employed in the first-line treatment of childhood ALL since the early 2000s in developed countries. These different formulations can be administered via intramuscular or intravenous routes and they possess similar mechanisms of action towards depleting L-asparagine in ALL patient’s bloodstream. However, a significant difference is seen in their pharmacokinetic properties. It was demonstrated that each L-asparaginase preparation has a distinct half-life in the body. The native *E. coli*, pegylated *E. coli* and *Erwinia* L-asparaginases had half-lives of 1.28 days, 5.73 days and 0.65 days, respectively, when injected intramuscularly (10). The specific dose required, schedule of administration, route of administration and monitoring of the enzyme activity level for each L-asparaginase preparation would be significantly affected due to their individual pharmacokinetic properties and thus, differ from one another (11). This article aimed to describe the clinical implication of L-asparaginase hypersensitivity and review the types of antibodies and genetic polymorphisms contributing to it.

### Hypersensitivity of Asparaginase

The antibody is a protein that serves as the body’s immune defence mechanism and is generated by plasma cells upon exposure to foreign molecules or antigens and other pathogens (12). Because L-asparaginase drug originates from a bacterial source, it is considered foreign to the body cells after administration into ALL patients. Sompayrac (13) explained that the emergence of an allergic reaction in the human body is provoked by foreign or non-native molecules that fulfil the following characteristics: possess a molecular weight higher than 100 kDa and present with a regularly recurring structure as well as surface epitopes of non-human origin for activation of B-cell receptors. Clinically available L-asparaginase preparations fulfil the features mentioned above, and hence tend to elicit production of anti-asparaginase antibodies from the human immune system, resulting in clinical hypersensitivity reactions or subclinical inactivation of L-asparaginases during treatment in ALL patients (14).

Antibodies produced will act against the antineoplastic drug, causing hypersensitivity reactions that clinically manifest as allergic reaction of varying degrees or subclinical hypersensitivity (silent inactivity) (15). Other than the adverse reaction, it also causes a reduction in L-asparaginase drug efficacy, leading to poor overall disease outcomes in paediatric ALL patients. Clinical hypersensitivity reaction was observed in 30% of patients administered with native *E. coli* L-asparaginase, and in 15% of those receiving pegylated *E. coli* and native *Erwinia* L-asparaginases (10). Formation of anti-asparaginase antibodies without clinical manifestation of hypersensitivity was observed in 8%–44% of the patients (16).

Identification of hypersensitivity events towards L-asparaginases becomes crucial in cases with subclinical hypersensitivity, which are usually clinically undetected. *Erwinia* L-asparaginase is antigenically distinct from *E. coli-*based formulation. Hence, there is cross immunoreactivity between the native *E. coli* L-asparaginase and its pegylated formulation, but not with the *Erwinia* asparaginase. In this paper, emphasis is placed on reviewing published information and data on hypersensitivity caused by anti-asparaginase antibodies in children with ALL.

**Figure 2:**
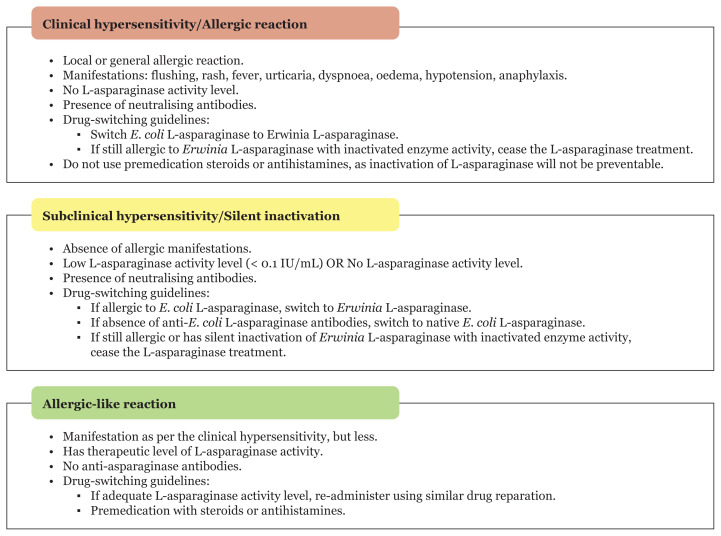
Classification of L-asparaginase hypersensitivity and their respective guidelines for switching between drug preparations

The Ponte di Legno Toxicity Working Group has classified L-asparaginase hypersensitivity into three major categories, as illustrated in Figure 2 (17, 18). Owing to the different hypersensitivity conditions and their manifestations in paediatric ALL patients, guidelines have been proposed by clinical physicians and researchers when switching between distinct L-asparaginase formulations, in search of a better L-asparaginase treatment outcome for the patients. The exact underlying mechanisms and molecular pathways for antibody production against L-asparaginase during chemotherapy in ALL patients are yet to be fully understood. Furthermore, L-asparaginase clinical hypersensitivity in response to antibodies has been reported to be influenced by several factors, such as patient age, formulation or preparation of L-asparaginase, the frequency of dosing and stages of chemotherapy (19–24).

Antibodies in humans are classified into five major isotypes: immunoglobulin (Ig)M, IgG, IgD, IgA and IgE, and all are synthesised from B lymphocytes or plasma cells. Existing publications reveal IgG, IgM and IgE to be the most frequently studied antibodies for L-asparaginase clinical hypersensitivity and silent inactivation (Table 1) (25–29).

There are substantial dissimilarities among IgG, IgM and IgE antibodies. Notably, IgM is the first antibody to be expressed in response to new exposure to pathogens, owing to its ability to be generated without very recent isotype switching. These antibodies exist mainly in blood and lymph. The molecular structure of IgM is made up of five interlinked Y-shaped monomers, forming a pentameric structure with 10 antigen-binding sites (therefore has a larger size), possessing a higher overall avidity yet a lower affinity for antigen binding. As for the antibodies IgG and IgE, both are present in monomeric structures, thus having a smaller size and capable of binding to the antigens with a higher affinity than IgM. IgG antibodies constitute approximately 75% of the human body’s total antibodies. They are found abundantly in the blood and extracellular fluid and primarily carry out opsonisation and neutralisation of antigens. On the other hand, IgE antibodies play a vital role in allergic reactions by binding to the mast cell receptors after binding to their respective antigens. Binding of IgE to mast cell receptors triggers mast cell activation which subsequently release the chemical mediators to expel the antigens, either through coughing, sneezing or vomiting (30).

Contradictory reports exist on the role of IgE antibodies in contributing to L-asparaginase hypersensitivity in paediatric ALL patients. Unlike IgE antibodies, established supportive data in the form of enzyme-linked immunosorbent assay (ELISA) studies, demonstrate the tendency of both, IgG and IgM antibodies to act against L-asparaginases in the serum or plasma of paediatric ALL patients (31). Some researchers claimed that IgE antibodies did not affect L-asparaginase hypersensitivity or the overall survival rates in children with ALL (25, 32). Nonetheless, Rathod et al. (33) had performed a novel study using murine models and discovered that specific IgG and IgE anti-asparaginase antibodies could potentially mediate L-asparaginase hypersensitivity in mice. Besides, they also found that FcγRIII receptors on IgG antibodies and FcɛRI receptors on IgE antibodies may play a role in generating hypersensitivity in mice immunised with L-asparaginase. Further studies are needed to identify the involvement of IgE antibodies in hypersensitivity events arising upon L-asparaginase administration in paediatric ALL patients.

**Figure 3:**
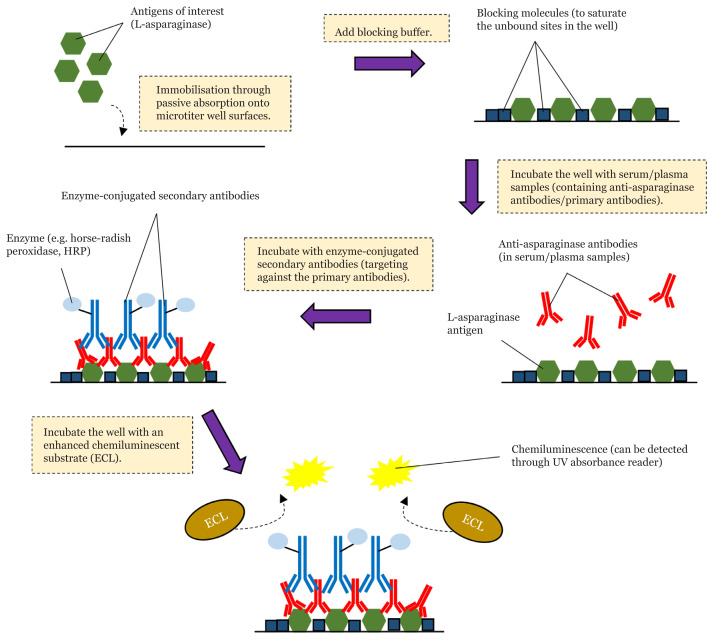
General steps of indirect ELISA assay establishment

Based on literature review, the indirect ELISA technique is seen to be the most popular and widely used quantification method for anti-asparaginase antibodies (IgG or IgM) using patient serum or plasma. Figure 3 illustrates the general steps of an indirect ELISA assay that can be used to identify anti-asparaginase antibodies in the serum or plasma samples (34, 35). Interestingly, apart from the indirect ELISA technique, some researchers had recently developed an alternative method using surface plasmon resonance (SPR) biosensor for detecting and quantifying serum anti-asparaginase antibodies in patients with ALL who underwent L-asparaginase treatment regimen. This technique can compensate for the shortcomings of the ELISA assay such as it being laborious, more time-consuming and less cost-effective, when analysing a small set of samples (36).

### Genetic Variants and Predisposition to L-Asparaginase Hypersensitivity

Pharmacogenetic studies have significantly contributed to the understanding of the different inter-patient variations in clinical and subclinical hypersensitivity development towards L-asparaginase during their cancer treatment. Identification of genetic variants can predict the predisposition of the paediatric ALL patients in developing L-asparaginase resistance or adverse reactions, helping establish a more suitable and rational treatment regimen individually tailored for each of the patients, in turn maximising the efficacy of the antineoplastic agent (37). The polymorphic genes contributing to L-asparaginase hypersensitivity have been studied and summarised in Table 2 (38–46).

Among the findings illustrated in Table 2, most of the studies were conducted in Caucasian paediatric ALL patients, while studies on Asians were rarely seen. Closure of such a knowledge gap can enable the potential establishment of a more thorough genetic variant monitoring protocol, prior to deciding on a treatment regimen. Table 3 depicts the major and minor allele frequencies among Asian populations for the single nucleotide polymorphisms. Their minor alleles were found to be associated with a higher risk of L-asparaginase hypersensitivity, except for those variants in linkage disequilibrium and tandem repeat polymorphism.

Recently, when studying the extent of hypersensitivity reaction in murine models towards L-asparaginase wherein the *NFATC2* gene was inhibited led to the discovery of a new variant of the gene (47). Previously, almost none of the published research findings included preclinical animal studies, thus resulting in a lack of information regarding the potential of gene knock-out or inhibitory interventions in guarding against L-asparaginase hypersensitivity. This research discovered that mice with an inactivated *NFATC2* gene did not develop hypersensitivity towards L-asparaginase after being immunised with the foreign enzyme. Moreover, the levels of IgE in these *NFATC2*-deficient mice were relatively lower than that in the wild-type mice. The researchers claimed that such gene inhibition outcomes might provide greater insight into generating attenuation against L-asparaginase hypersensitivity in human subjects in the near future (47). More studies are required to understand further the mechanisms of action of the various genetic variants in predisposing children with ALL to greater risk of having hypersensitivity against L-asparaginase.

## Conclusion

L-asparaginase hypersensitivity has long been a deterrent in improving the treatment outcome, and the survival rates of children suffering from ALL. Clinicians and medical researchers around the world have made significant efforts towards understanding the roles of anti-asparaginase antibodies and the identification of genetic variants related to it in the hope of addressing L-asparaginase hypersensitivity. The mechanisms of action of how these antibodies target L-asparaginase after the drug administration as well as the specific pathways involved in these reactions remain unclear till date and need to be studied in future. Based on current understanding, further and more thorough studies are anticipated to help optimise the therapy in paediatric ALL patients.

## Figures and Tables

**Table 1:** Summaries of literature findings regarding anti-L-asparaginase antibodies in paediatric ALL patients

Authors, year	Target anti-L-asparaginase antibody sub-type(s)	Study details	Findings in brief	Ref.
Galindo-Rodríguez et al., 2017	IgG IgE	Cross-sectional study. Cohort: 51 paediatric patients (below 16 years old) with ALL of precursor B-cell origin. IgG quantification method: ELISA. IgE detection method: Intradermal and prick skin testing.	Ten patients with only IgG; 18 patients with only IgE; 14 patients with both IgG and IgE. ALL patients with IgG against L-asparaginase were presented with higher rate of relapse. ALL patients with IgE after first relapse case did not develop additional relapses.	(25)
Tong et al., 2014	IgG IgM	Prospective study. Cohort: Paediatric ALL patients (1 year old– 18 years old) administered with PEG-asparaginase or *Erwinia* asparaginase. IgG quantification method: ELISA IgM quantification method: ELISA	A correlation was found between presence of asparaginase antibodies and occurrence of allergic reactions or subclinical hypersensitivity. However, there was a low specificity (64%) for the antibody ELISA test.	(26)
Liu et al., 2012	IgG	Cohort: 410 paediatric patients with ALL and receiving native *E. coli* asparaginase, pegylated asparaginase, or *Erwinia* asparaginase. Quantification method: ELISA	Patients exhibiting allergic reaction to native *E. coli* asparaginase (Elspar) were found to more frequently have the IgG antibodies than those without clinical allergy.	(27)
Woo et al., 2000	IgG	Cohort: 154 children with newly diagnosed ALL. Quantification method: ELISA	A total of 54 patients developed anti-Elspar IgG. Among them, 56% showed clinical hypersensitivity reaction, while 44% of them did not have allergy. A total of 98 patients showed absence of anti-Elspar IgG. Among them, only 18% showed clinical hypersensitivity reaction. However, no correlation was found between presence of anti-asparaginase antibodies and treatment outcome in the patients.	(28)
Zalewska-Szewczyk et al., 2007	IgG IgM	Prospective study. Cohort: 47 paediatric patients with newly diagnosed ALL. IgG quantification method: ELISA IgM quantification method: ELISA	Patients possessing anti-asparaginase antibodies had lower asparaginase activity level. There was an association between clinical hypersensitivity and anti-asparaginase antibodies. Lower event-free survival and overall survival was discovered in patients with anti-asparaginase antibodies during induction phase, compared to those without the antibodies.	(29)

Notes: ALL = acute lymphoblastic leukaemia; ELISA = enzyme-linked immunosorbent assay; IgG = immunoglobulin G; IgM = immunoglobulin M; IgE = immunoglobulin E

**Table 2:** Summaries of genetic variants with significant association to asparaginase hypersensitivity in paediatric patients

Author, year	Study cohort (*n*)	Method	Asparaginase preparation in study cohort	Gene	SNP	Genetic variant	Ref.
Rajić et al., 2015	146	SNPs genotyping	Native *E. coli*; pegylated *E. coli; Erwinia* asparaginases	GRIA1	rs4958351	G>A	(38)
					rs10070447	C>T	
					rs6890057	C>T	
					rs4958676	G>A	
					rs6889909	C>T	
Ben Tanfous et al., 2015	285	SNPs genotyping	*E. coli* asparaginase	ASNS	rs3832526	3R3R (Tandem repeat polymorphism)	(39)
Højfeldt et al., 2019	831	GWAS	Pegylated *E. coli* asparaginase	CNOT3	rs73062673	T>C	(40)
				HLA_DQA1	rs9272131	C>T	
				TAP2	rs115360810	A>G	
Fernandez et al., 2015	3308	GWAS	Native and pegylated *E. coli* asparaginases	NFATC2	rs6021191	A>T	(41)
				HLA_DRB1	rs17885382	C>T	
Chua et al., 2021	107	SNPs genotyping	Native *E. coli*; pegylated *E. coli; Erwinia* asparaginases	HLA_B*46:01	-	-	(42)
				HLA_DRB1*09:01	-	-	
Liu et al., 2021	4259	GWAS	Pegylated *E. coli* asparaginase	HLA_DQB1*02:02	-	-	(43)
				HLA_DRB1*07:01	-	-	
				HLA_DQA1*02:01	-	-	
Gagné et al., 2020	284	EWAS, SNPs genotyping	Native *E. coli* asparaginase	HLA_DQB1*07:01-DQB1*02:02	-	-	(44)
Abaji et al., 2017	282	EWAS	Native *E. coli*; pegylated *E. coli; Erwinia* asparaginases	SLC7A13	rs9656982	A>G	(45)
				MYBBP1A	rs3809849	G>C	
				YTHDC2	rs75714066	G>C	
Chen et al., 2010	322	GWAS	N/A	GRIA1	rs4958351	G>A	(46)

Notes: SNP = single nucleotide polymorphism; GWAS = genome-wide association study; EWAS = exome-wide association study

**Table 3:** SNPs allele frequencies in Asian populations (retrieved from NCBI SNP database)

Gene	Position	SNP	Major > minor allele	Population	Major allele frequency	Minor allele frequency
*GRIA1*	Chromosome 5	rs4958351	G > A	Asian	0.948	0.052
				East Asian	0.983	0.017
				South Asian	0.484	0.516
				Other Asian	0.88	0.12
		rs10070447	C > T	Asian	0.944	0.056
				East Asian	0.990	0.010
				South Asian	0.500	0.500
				Other Asian	0.86	0.14
		rs6890057	C > T	Asian	0.983	0.017
				East Asian	0.995	0.005
				South Asian	0.848	0.152
				Other Asian	0.960	0.040
		rs4958676	G > A	Asian	0.975	0.025
				East Asian	0.988	0.012
				South Asian	0.7963	0.2037
				Other Asian	0.95	0.05
		rs6889909	C > T	Asian	0.963	0.037
				East Asian	0.990	0.010
				South Asian	0.864	0.136
				Other Asian	0.91	0.09
*CNOT3*	Chromosome 19	rs73062673	T > C	Asian	0.971	0.029
				East Asian	0.972	0.028
				South Asian	0.746	0.254
				Other Asian	0.96	0.04
*HLA_DQA1*	Chromosome 6	rs9272131	C > T	Asian	0.936	0.064
				East Asian	0.938	0.062
				South Asian	0.921	0.079
				Other Asian	0.93	0.07
*TAP2*	Chromosome 6	rs115360810	A > G	Asian	1.0000	0.0000
				East Asian	1.0000	0.0000
				South Asian	1.0000	0.0000
				Other Asian	1.0000	0.0000
*NFATC2*	Chromosome 20	rs6021191	A > T	Asian	0.929	0.071
				East Asian	0.93	0.07
				South Asian	0.98	0.02
				Other Asian	0.92	0.08
*HLA_DRB1*	Chromosome 6	rs17885382	C > T	Asian	0.964	0.036
				East Asian	0.97	0.03
				South Asian	0.90	0.10
				Other Asian	0.96	0.04
*SLC7A13*	Chromosome 8	rs9656982	A > G	Asian	0.8556	0.1444
				East Asian	0.8596	0.1404
				South Asian	0.8403	0.1597
				Other Asian	0.8456	0.1544
*MYBBP1A*	Chromosome 17	rs3809849	G > C	Asian	0.919	0.081
				East Asian	0.93	0.07
				South Asian	1.00	0.00
				Other Asian	0.90	0.10
*YTHDC2*	Chromosome 5	rs75714066	G > C	Asian	0.9275	0.0725
				East Asian	0.9158	0.0842
				South Asian	0.938	0.062
				Other Asian	0.975	0.025

Note: SNPs = single nucleotide polymorphisms
